# Risk of Neonatal Sepsis With Rescue Steroids in Preterm Premature Rupture of Membranes

**DOI:** 10.7759/cureus.37207

**Published:** 2023-04-06

**Authors:** Emily Tenbrink, Angela Quain, Victoria Rone, Kate Harris, Emily Hadley, David Haas, Anthony Shanks

**Affiliations:** 1 Obstetrics and Gynecology, Indiana University School of Medicine, Indianapolis, USA

**Keywords:** preterm premature rupture of membranes, rescue steroids, obstetrics & gynecology, premature prolonged rupture of membranes, neonatal sepsis, antenatal steroids

## Abstract

Objective

To evaluate whether a rescue course of corticosteroids, when given at least 14 days after the initial course, is associated with an increased risk of neonatal sepsis after preterm premature rupture of membranes (PPROM).

Methods

We performed a retrospective, descriptive cohort study of women with singleton gestations from 23+0 to 34+0 weeks of gestation who received a rescue course of corticosteroids within the Indiana University Health Network from January 2009 through October 2016. Patients were separated into three groups based on amniotic membrane status at the time of each corticosteroid administration: Group 1 (intact membranes at initial/intact membranes at rescue), Group 2 (intact membranes at initial/PPROM at rescue), and Group 3 (PPROM at initial/PPROM at rescue). The primary outcome (neonatal sepsis) was compared between the groups. Patient characteristics and neonatal outcomes were analyzed with Fisher’s exact test for categorical variables and ANOVA for continuous variables. Relative risk (RR) was calculated by comparing those with ruptured membranes to those with intact membranes at the time of rescue course administration.

Results

A total of 143 patients were eligible. Neonatal sepsis occurred in 6.8% of patients in Group 1, 21.1% of patients in Group 2, and 23.8% of patients in Group 3. Groups 2 and 3 had a statistically significant higher rate of neonatal sepsis than Group 1 (p = 0.021). The RR of neonatal sepsis after a rescue course in patients with PPROM (Groups 2 and 3) was 3.31 (95% CI = 1.32, 8.29) compared to those with intact membranes at the time of rescue course administration (Group 1).

Conclusion

A rescue course of corticosteroids in women with PPROM at the time of rescue administration was associated with an increased risk of neonatal sepsis. This increased risk was seen in women with intact membranes as well as ruptured membranes during their initial course of steroids. Larger studies are needed to further investigate this association.

## Introduction

The administration of antenatal corticosteroids to women at risk for preterm birth is one of the most important interventions to improve neonatal outcomes. A single course of antenatal corticosteroids administered to mothers who deliver prematurely (less than 34 weeks) can reduce the risk of mortality, respiratory distress syndrome (RDS), and intraventricular hemorrhage (IVH) in the neonate [1]. The benefits of corticosteroid administration are observed between 48 hours and seven days after administration [1-3]. Given the waning benefits after one week and the imprecision of identifying patients with preterm labor who ultimately deliver preterm, the administration of weekly steroids was investigated. This practice of multiple courses of corticosteroids was further justified by several studies that found an improvement in neonatal RDS after multiple courses [4,5]. However, neonates treated with multiple courses of corticosteroids had a significant decrease in birth weight and head circumference, as well as an increase in neonatal morbidity [6-9]. Furthermore, studies found that multiple doses of corticosteroids in patients with preterm premature rupture of membranes (PPROM) were associated with an increased risk of neonatal sepsis [10,11]. Based on these findings, the recommendation by the National Institutes of Health at that time was a single course of antenatal corticosteroids in women at risk of preterm delivery [12].

In 2009, Garite et al. performed a multicenter, randomized, double-blind, placebo-controlled trial assessing neonatal outcomes after a single rescue course of corticosteroids in patients at risk for preterm birth. A "rescue course" in this study was defined as a repeat course of corticosteroids in patients at risk for preterm birth after at least 14 days had passed since the initial course was given. The results demonstrated a significant decrease in neonatal morbidity and RDS and no significant negative effects when a single rescue course of corticosteroids was administered [13]. Notably, this study did not include PPROM patients; therefore, the risks and benefits of a rescue course of corticosteroids in the setting of PPROM remain unclear. Thus, the objective of this study is to evaluate whether a rescue course of corticosteroids, when given at least 14 days after the initial course, is associated with an increased risk of neonatal sepsis after PPROM.

This study was presented at The American College of Obstetricians and Gynecologists Annual Meeting in Austin, Texas on April 28, 2018.

## Materials and methods

We performed a retrospective, descriptive cohort study of pregnant women with singleton gestations from 23+0 to 34+0 weeks of gestation who received a rescue course of antenatal corticosteroids within the Indiana University Health Network from January 2009 through October 2016. A rescue course of corticosteroids was defined as a single course of corticosteroids administered a minimum of 14 days after the initial course. For the duration of this study, all pregnant women with gestations from 23+0 to 34+0 weeks of gestation were offered rescue steroids. The study was approved by the institutional review board at our institution as a retrospective protocol. Study data were collected and managed using REDCap (Research Electronic Data Capture; Vanderbilt University, Nashville, TN) electronic data capture tools hosted at Indiana University School of Medicine [14,15]. REDCap is a secure, web-based software platform designed to support data capture for research studies, providing (1) an intuitive interface for validated data capture, (2) audit trails for tracking data manipulation and export procedures, (3) automated export procedures for seamless data downloads to common statistical packages, and (4) procedures for data integration and interoperability with external sources.

Subjects were identified by pharmacy codes for four total doses of betamethasone (12 mg intramuscular (IM) every 24 hours for four orders) or eight total doses of dexamethasone (6 mg IM every 12 hours for eight orders) during a pregnancy. Additionally, subjects were identified using the keywords “rescue steroids” or “rescue betamethasone” in admission history and physicals, and discharge summaries. Exclusion criteria included subjects with major fetal anomalies, stillbirth, multiple gestations, active maternal infection, delivery outside of the hospital system (inaccessible delivery records), and women who received more than two courses of corticosteroids.

Medical records were reviewed to obtain maternal demographic data and maternal comorbidities. Gestational age was established according to the earliest available sonogram in the electronic medical record. PPROM was diagnosed if two of the following four criteria were met: (1) pooling of amniotic fluid in the vaginal vault, (2) positive nitrazine test, (3) positive ferning of vaginal fluid, or (4) positive ROM Plus® test.

Patients were separated into three groups based on amniotic membrane status at the time of corticosteroid administration. Group 1 included patients who received their initial course and rescue course of steroids with intact membranes. Group 2 included patients who received their initial course of steroids with intact membranes and their rescue course of steroids with ruptured membranes. Group 3 included patients who received both courses of steroids with ruptured membranes.

Neonatal records were reviewed to obtain delivery and outcome data. Our primary outcome was neonatal sepsis, defined as positive culture (urine, blood, sputum, or cerebral spinal fluid) and infection suspected on physical exam. Or, in the absence of positive cultures, neonatal sepsis was defined as clinically suspected sepsis with vital sign abnormalities (fever, hypotension, tachycardia) and X-ray findings. Secondary outcomes included one-minute and five-minute Apgar scores, birth weight, and diagnosis of RDS, IVH, and necrotizing enterocolitis (NEC).

Baseline demographics and characteristics were compared using Fisher’s exact test for categorical variables and ANOVA for continuous variables. Neonatal outcomes were also compared using Fisher’s exact test for categorical variables and ANOVA for continuous variables. Statistical significance was defined as p < 0.05. Relative risk was calculated by comparing those with ruptured membranes to those with intact membranes at the time of rescue course administration. Statistical analyses were conducted using SAS V9.4 (SAS Institute Inc., Cary, NC).

## Results

**Figure 1:**
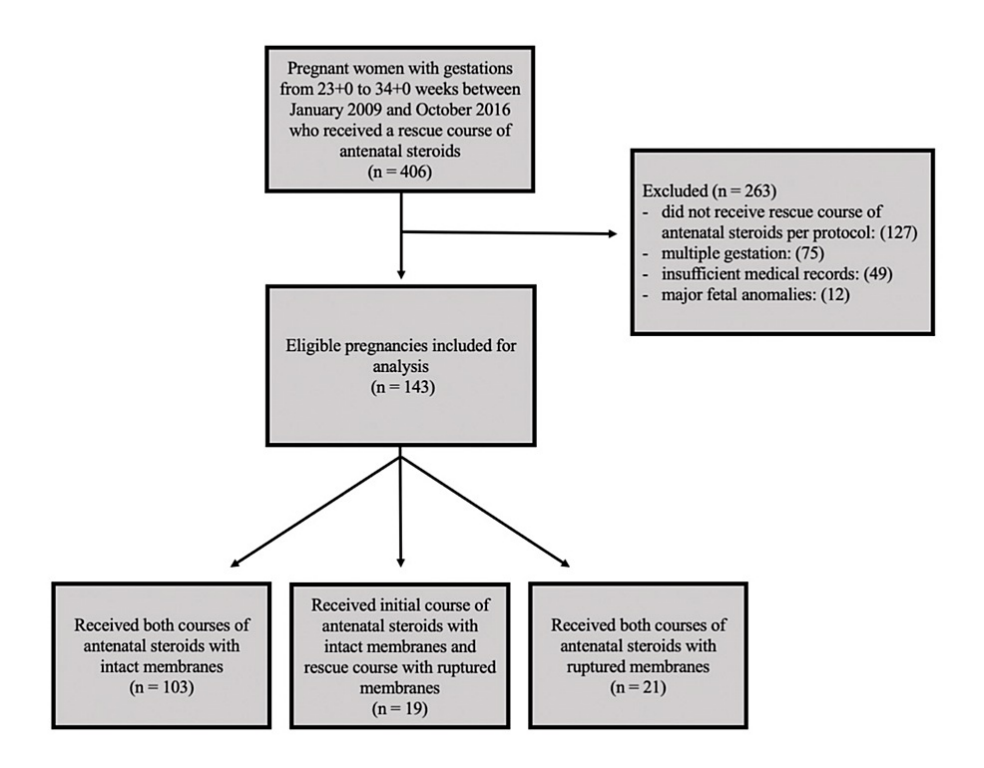
Flowchart of patients included in the study

Using the search criteria listed above, 406 patients were identified. A total of 263 patients were excluded; 127 due to not receiving a rescue course of antenatal corticosteroids as defined above, 75 due to multiple gestations, 49 due to insufficient maternal or neonatal medical records, and 12 due to major fetal anomalies. A total of 143 patients met the inclusion criteria for analysis (Figure 1).

A total of 103 patients received both their initial course and their rescue course of steroids with intact membranes (Group 1). A total of 19 patients received their initial course of steroids with intact membranes and their rescue course of steroids with ruptured membranes (Group 2). A total of 21 patients received both their initial course and their rescue course of steroids with ruptured membranes (Group 3). Though both betamethasone and dexamethasone were queried for as described in the methods section, all patients who met inclusion criteria for analysis had received betamethasone 12 mg IM every 24 hours for four orders for both courses.

No statistically significant differences were found between the three groups in terms of age, race, Group B streptococcal status, hypertensive disease, diabetes, chronic steroid use, exposure to magnesium sulfate, mode of delivery, or sex of the neonate (Tables 1, 2). Group 3 had a statistically significant lower use of tobacco (p = 0.035) and lower BMI (p = 0.005) than Groups 1 and 2. Patients in Group 2 received tocolysis more than the other two groups (p = 0.007). Patients in Groups 2 and 3 received latency antibiotics more often than Group 1 (p < 0.001).

**Table 1:** Maternal clinical and demographic data

Outcome	Overall	Group 1	Group 2	Group 3	P-value
Race (%)					
Asian	2 (1.4)	1 (1.0)	1 (5.3)	0 (0.0)	
Black or African American	42 (29.4)	32 (31.1)	3 (15.8)	7 (33.3)	
Native Hawaiian or Other Pacific	1 (.7)	1 (1.0)	0 (0.0)	0 (0.0)	
White	93 (65.0)	65 (63.1)	14 (73.7)	14 (66.7)	
Unknown/not reported	5 (3.5)	4 (3.9)	1 (5.3)	0 (0.0)	
Age (years)	28.2 ± 6.1	28.3 ± 5.9	28.4 ± 7.3	27.4 ± 6.1	0.808
BMI	32.3 ± 8.2	33.6 ± 8.2	30.2 ± 8.5	27.6 ± 5.8	0.005
Tobacco use (%)	47 (34.3)	37 (36.6)	8 (47.1)	2 (10.5)	0.035
Group B beta strep (%)					
Positive	38 (27.0)	26 (25.7)	5 (26.3)	7 (33.3)	0.292
Unknown	35 (24.8)	30 (29.7)	2 (10.5)	3 (14.3)	
Hypertension (%)					
Chronic hypertension	12 (8.4)	11 (10.7)	1 (5.3)	0 (0.0)	0.201
Gestational hypertension	6 (4.2)	6 (5.8)	0 (0.0)	0 (0.0)	
None	103 (72.0)	67 (65.0)	16 (84.2)	20 (95.2)	
Pre-eclampsia	22 (15.4)	19 (18.4)	2 (10.5)	1 (4.8)	
Diabetes (%)					
Type 1 diabetes	3 (2.1)	2 (1.9)	0 (0.0)	1 (4.8)	0.344
Type 2 diabetes	6 (4.2)	6 (5.8)	0 (0.0)	0 (0.0)	
Gestational diabetes	16 (11.2)	14 (13.6)	2 (10.5)	0 (0.0)	
None	118 (82.5)	81 (78.6)	17 (89.5)	20 (95.2)	
Chronic steroid use (%)	2 (1.4)	2 (1.9)	0 (0.0)	0 (0.0)	>0.999
Magnesium sulfate (%)	71 (50.7)	48 (47.1)	11 (61.1)	12 (60)	0.395
Tocolysis (%)					
Indomethacin	12 (8.5)	8 (7.8)	2 (10.5)	2 (9.5)	0.007
Magnesium sulfate	18 (12.7)	10 (9.8)	1 (5.3)	7 (33.3)	
Nifedipine	26 (18.3)	19 (18.6)	7 (36.8)	0 (0.0)	
None	86 (60.6)	65 (63.7)	9 (47.4)	12 (57.1)	
Latency antibiotics	46 (32.2)	7 (6.8)	18 (94.7)	21 (100.0)	<0.0001
Mode of delivery (%)					
Cesarean section	79 (55.2)	63 (61.2)	7 (36.8)	9 (42.9)	0.066
Vaginal	64 (44.8)	40 (38.8)	12 (63.2)	12 (57.1)	
Chorioamnionitis (%)	8 (5.6)	1 (1.0)	5 (26.3)	2 (9.5)	<0.001>

**Table 2:** Gestational age at steroid course administration

Outcome		Overall	Group 1	Group 2	Group 3	P-value
Gestational age at initial steroid course		26.8 ± 2.8	27.2 ± 2.9	26.5 ± 2.1	25.3 ± 2.4	0.017
Gestational age at rescue steroid course		30.8 ± 2.6	31.2 ± 2.5	30.6 ± 2.7	29.1 ± 2.9	0.003

Neonatal sepsis was diagnosed more frequently in patients with PPROM at the time of their rescue course compared to those with intact membranes at the time of their rescue course (Table 3). Sepsis was diagnosed in 21.1% of Group 2 patients and 23.8% of Group 3 patients compared to 6.8% in Group 1 (Table 4). This difference was statistically significant (p = 0.021). The relative risk of neonatal sepsis after a rescue course of steroids in women with PPROM was 3.31 (95% CI = 1.32, 8.29) compared to those with intact membranes.

**Table 3:** Odds ratio and relative risk of neonatal sepsis with rescue steroid administration

Patient cohort	Odds ratio (95% CI)	Relative risk (95% CI)
Intact 1^st^/ruptured 2^nd^ vs. intact	3.66 (0.95-14.02)	3.10 (1.00-9.56)
Ruptured 1^st^ and 2^nd^ vs. intact	4.29 (1.21-15.17)	3.50 (1.23-9.98)

**Table 4:** Neonatal outcomes with rescue steroid administration

Outcome	Overall	Group 1	Group 2	Group 3	P-value
Neonatal sepsis (%)	16 (11.2)	7 (6.8)	4 (21.1)	5 (23.8)	0.021
Respiratory distress syndrome (%)	78 (54.5)	47 (45.6)	12 (63.2)	19 (90.5)	<0.001
Doses of surfactant (%)					
0	41 (59.4)	25 (64.1)	7 (63.6)	9 (47.4)	
1	17 (24.6)	10 (25.6)	3 (27.3)	4 (21.1)	
2	9 (13.0)	3 (7.7)	1 (9.1)	5 (26.3)	
3	1 (1.4)	0 (0.0)	0 (0.0)	1 (5.3)	
4	1 (1.4)	1 (2.6)	0 (0.0)	0 (0.0)	
Necrotizing enterocolitis (%)	3 (2.1)	1 (1.0)	1 (5.3)	1 (4.8)	0.194
Intraventricular hemorrhage (%)	5 (3.5)	2 (2.0)	1 (5.3)	2 (9.5)	0.136
Neonatal death (%)	4 (2.8)	3 (2.9)	0 (0.0)	1 (4.8)	0.738
Neonate days in the hospital	37.8 ± 34.4	32.3 ± 34.4	44.2 ± 29.2	59.1 ± 30.5	0.003

No significant difference was found between the three groups in the mean time between their first course and rescue course of steroids (Group 1 - mean weeks between courses: 4.06, SD: 2.4 weeks; Group 2 - mean weeks between courses: 4.05, SD: 2.09 weeks; Group 3 - mean weeks between courses: 3.7, SD: 1.9 weeks; p = 0.80). Group 3 had a significantly lower mean gestational age at the time of the initial steroid course (25.3 ± 2.4 weeks) and rescue course (29.1 ± 2.9 weeks) compared to Group 2 (26.5 ± 2.1 weeks initial and 30.6 ± 2.7 weeks rescue) and Group 1 (27.2 ± 2.9 weeks initial and 31.2 ± 2.5 weeks rescue) (p = 0.017 for initial course and p = 0.003 for rescue course).

A significantly greater rate of chorioamnionitis was appreciated among patients in Group 2 (p < 0.001). Group 3 had a significantly greater rate of neonatal RDS (90.5%) compared to Group 1 (45.6%) and Group 2 (63.2%) (p < 0.001). No significant difference in the rate of neonatal NEC or IVH was found between the three groups. Group 3 had a significantly lower mean gestational age at the time of delivery (p < 0.001) as well as a significantly lower mean birth weight (p < 0.001) and a significantly higher mean number of days in the hospital for the neonate (p < 0.003). No significant difference was found in neonatal death and Apgar scores at one and five minutes between the three groups (Table 5).

**Table 5:** Neonatal delivery demographic data

Outcome	Overall	Group 1	Group 2	Group 3	P-value
Gestational age at delivery	32.7 ± 3.4	33.6 ± 3.2	31.3 ± 2.7	29.6 ± 2.8	<0.0001
1-minute Apgar	6.5 ± 2.4	6.6 ± 2.5	6.5 ± 2.5	6.3 ± 2.0	0.874
5-minute Apgar	7.9 ± 1.6	8.0 ± 1.6	8.3 ± 1.2	7.2 ± 1.5	0.052
Birth weight	1894.8 ± 769.1	2057.2 ± 792.7	1610.5 ± 479.6	1355.3 ± 518.9	<0.0001
Live birth	143 (100.0)	103 (100.0)	19 (100.0)	21 (100.0)	
Sex of neonate (%)					
Female	62 (44.9)	45 (45.5)	8 (42.1)	9 (45.0)	>0.999
Male	76 (55.1)	54 (54.5)	11 (57.9)	11 (55.0)	

## Discussion

In our retrospective, descriptive cohort study of women with singleton gestations from 23+0 to 34+0 weeks of gestation who received a rescue course of antenatal corticosteroids, defined as a course of corticosteroids being administered after a minimum of 14 days from the initial course, women with PPROM at the time of rescue steroid administration had an increased risk of neonatal sepsis. This increased risk was irrespective of membrane status at the time of their initial course of steroids. Furthermore, the rate of neonatal RDS was not decreased when rescue steroids were administered to women with PPROM. In fact, women with PPROM at the time of both the initial course and the rescue course of steroids had neonates with a significantly greater rate of RDS, lower birth weight, and longer length of stay in the hospital.

Prior studies have attempted to evaluate the risk of neonatal sepsis when patients with PPROM were administered a repeat course of corticosteroids. One study demonstrated no difference in neonatal sepsis rates when comparing one course (16% risk of neonatal sepsis) with two courses (17.2% neonatal sepsis, p = 0.76) [16]. However, the overall rates of neonatal sepsis in this study were higher when compared to historical controls and the repeat course utilized differed from contemporary recommendations [13].

A noteworthy strength of our study is that it was performed at a single institution with standardized care of patients with preterm labor and PPROM. Our study was able to account for the type of corticosteroid administered (all patients received the same steroid) and standardize the length of time between corticosteroid courses (a minimum of 14 days). Another strength of our study was the ability to link maternal and neonatal outcomes.

This study contains important limitations that warrant discussion. The small sample size of 143 patients causes our study to be underpowered to observe differences in some outcomes. Also, given that this is a retrospective, descriptive cohort study, no causality can be attributed to our findings as our study did not include a comparison to women who received an initial steroid dose but did not receive a rescue dose after PPROM. One potential explanation for the increased risk of neonatal sepsis in our patients with ruptured membranes at the time of rescue course administration is that women with PPROM are more likely to be exposed to potentially pathologic microorganisms than women with intact membranes [17]. However, given that the time frame between the initial and rescue courses was similar for all three groups, a significant increase in antepartum infection alone is unlikely to be the sole reason for the increase in neonatal sepsis. Though the mean time between administration of initial steroids and rescue steroids did not differ per group, the gestational age of delivery did differ, which may impact results.

## Conclusions

In conclusion, our findings demonstrate that a rescue course of corticosteroids in women with PPROM was associated with an increased risk of neonatal sepsis. This increased risk was seen in women with intact membranes as well as ruptured membranes at the time of their initial course of steroids. Additionally, the use of rescue steroids in women with PPROM was not associated with a reduction in the rate of neonatal RDS. Larger studies are needed to further investigate this observed association.
